# Conserved host genes act as anchors for fast-evolving piRNA clusters via promoter hijacking

**DOI:** 10.21203/rs.3.rs-9860925/v1

**Published:** 2026-06-05

**Authors:** Aleksandr Friman, Anand Swaroop

**Affiliations:** 1 Neurobiology, Neurodegeneration & Repair Laboratory, National Eye Institute, National Institutes of Health, 6 Center Dr, Bethesda, 20892, Maryland, USA.; 2 Biophysics Graduate Program, University of Maryland, 8108 Regents Dr, College Park, 20742, Maryland, USA.

**Keywords:** piRNA, PIWI, transposon, spermatogenesis

## Abstract

PiRNA clusters evolve rapidly but are strictly regulated at the same time. Previous reports have suggested a co-transcription mechanism using tight regulation of coding genes. To test whether the “Promoter Hijacking” hypothesis is a plausible mechanism, we developed a new method to identify gene orthologs in poorly annotated genomes of placental mammals and investigated co-transcription of stochastic piRNA clusters. Our method uses context aware Hidden Markov Models analysis allowing the resolution of ambiguities between paralogs using the nearby genes. We demonstrate that the identified genes are preferentially expressed in the testes, where the piRNA pathway is most active. Our findings suggest the existence of dual-function loci, where the host gene retains its primary biological function, i.e. the production of an essential transcript or protein, subjecting the locus to strong purifying selection and ensuring its genomic stability. Concurrently, the locus acquires a secondary immune function by the generation of a long, target-laden precursor transcript that feeds the piRNA biogenesis pathway. This secondary region remains free from protein-coding constraints, allowing it to rapidly accumulate and retain new target insertions. Thus, “Promoter Hijacking” mechanism effectively updates genomic immune memory without compromising the stability or function of the host locus.

## 1 Introduction

The eukaryotic germline is the immortal lineage of multicellular organisms, linking generations through an unbroken chain of genetic inheritance. However, this immortality comes at a cost since the germline and the zygote serve as crucial genomic bottlenecks, where mutations can lead to the heritable corruption of the organism. Mobile Genetic Elements (MGEs), including Transposable Elements (TEs) and Endogenous Retroviruses (ERVs), continually seek to replicate within the germline, threatening host fitness through insertional mutagenesis and chromosomal instability [1]. To suppress this threat, animals have evolved the PIWI-interacting RNA (piRNA) pathway, a small RNA-based immune system that functions as a genomic database of MGE sequence signatures [2].

The piRNA pathway operates on the principle of “self vs. non-self” recognition, guided by small RNAs derived from discrete genomic loci known as piRNA clusters. These clusters serve as the immune system’s memory banks—graveyards of fragmented MGE sequences that are transcribed, processed, and loaded onto PIWI-clade proteins to guide the silencing of active mobile elements. The key protein machinery of this pathway is highly conserved across metazoans, yet the piRNA clusters themselves are fast evolving to keep up with the MGEs evolution [3]. This rapid turnover presents a fundamental evolutionary paradox. How does the host genome regulate stable, reliable expression of the defense mechanism when the underlying genetic loci are inherently unstable and constantly shifting?

To date, evolutionary studies of the piRNA pathway have struggled to resolve this question, often limited by narrow phylogenetic scopes [4–6] or a heavy dependence on the sporadic availability of small RNA sequencing data [3]. To address this gap, we propose the “Promoter Hijacking” hypothesis. Borrowing a conceptual framework from cancer biology [7], this model is based on the opportunistic co-option of existing regulatory architecture, building upon prior observations of piRNA cluster co-transcription with a large Maf gene [8]. In our model, the intense evolutionary pressure to repress mobile elements drives the piRNA machinery to co-opt the robust promoters of stable host genes. Rather than evolving *de novo* regulatory mechanisms for every newly emerged piRNA cluster, the genome expands the functional utility of existing genes. By extending transcription beyond the canonical polyadenylation site and into the downstream intergenic space, the transcriptional apparatus of a host gene is “hijacked” to drive the expression of adjacent MGE-rich regions.

To date, only sporadic observations of such co-opted promoter-driven MGE suppressing loci have been reported, limited to a few specific examples such as the non-coding *Zim2* locus [9] and the protein-coding *NOPCHAP1* gene [10] in eutherian mammals.

In this study, we systematically test the Promoter Hijacking hypothesis through a comprehensive comparative genomics screen. Expanding on previous isolated observations, we analyzed 223 eutherian mammal genomes and identified orthologous host genes that have been systematically co-opted for this secondary immune function. By integrating hidden Markov model (HMM) profiling with a novel synteny-aware orthology detection pipeline, we identify a conserved set of “anchor” genes. These dual-function loci exhibit a striking evolutionary signature: deep conservation of the host gene sequence paired with a dynamic, strand-purified, MGE-rich downstream region that reliably fuels the piRNA response across diverse mammalian lineages. Our findings suggest that the integration of host physiology and genomic defense is far tighter than previously appreciated, with essential cellular factors serving as the immutable anchors for the rapidly evolving machinery of genome defense.

## 2 Methods

### 2.1 Genome Assembly and Annotation Acquisition

We obtained genomic data for 223 Eutherian mammal species from the NCBI Genomes database (https://www.ncbi.nlm.nih.gov/datasets/genome/). To ensure high-quality orthology prediction, we restricted our analysis to assemblies with available gene annotation (GTF/GFF3) and corresponding protein sequences (FASTA). As representative genomes we utilized the human genome (GRCh38.p13) and 8 other diverse mammalian species – mouse (*M. musculus*), dog (*C. lupus familiaris*), cat (*F. catus*), cow (*B. taurus*), camel (*C. dromedarius*), elephant (*L. Africana*), pig (*S. scrofa*), whale (*B. musculus*) – for which Ensembl [11] provides orthology with human genes.

### 2.2 Construction of bijective gene signatures

To track orthologous loci across diverse lineages, we generated a set of highly specific Hidden Markov Models (HMMs). Using Ensembl orthology annotation we selected the protein coding genes having only one ortholog between the human genome and each of the other representative species. For each of the bijective genes we extracted all protein-coding sequences from the representative genomes and constructed HMM profiles using MUSCLE [12] and hmmer v3.4 [13]. To minimize paralogous confounding, we subjected each model to self-consistency testing against the full proteomes of the nine representative genomes. A model was retained only if it exhibited high specificity, defined as generating no more than two false-positive hits.

### 2.3 Synteny-Aware Orthology Prediction

Sequence similarity alone is often insufficient for identifying orthologs in poorly annotated non-model assemblies. We therefore developed a context-aware identification pipeline using association rule mining.

#### 2.3.1 Hierarchical Target Identification

To validate ortholog identification, we backtested association rules against the coding genes of representative species using the following hierarchical approaches: **Fast Track (Exact Matches):** The hits mapping uniquely to the original human Ensembl ID were accepted immediately.

**Synteny Track (Ambiguous Matches):** For hits with ambiguous or multiple mappings, we queried the synteny database. A candidate was validated only if it satisfied more than 10 association rules (presence of required neighbors within 5 Mb) and the synteny score (number of satisfied rules) was at least 10 times higher than that of the second-best candidate.

For a gene to be further analyzed it should be detected in at least 50 genomes and in at least 10 taxonomic families with 3 analyzed genomes each.

### 2.4 Filtration of MGE-Derived Coding Sequences

We performed a targeted filtration step to prevent retroviral Open Reading Frames (ORFs) from being misclassified as host genes as this would lead to misidentification of the intergenic space boundaries, we performed a targeted filtration step. We curated a list of 48 InterPro domains associated with mobile elements (e.g., Integrase, Reverse Transcriptase, Gag) by searching for keywords “retrovirus”, “transpos”, and “poly-protein”, while explicitly retaining host functional domains (e.g., “Zinc finger”, “Kinase”). We scanned the identified orthologs using InterProScan (v5.72) [14]. Genes were excluded if they contained MGE-associated domain and possessed a generic identifier (e.g., LOC followed by at least 4 digits). At this step each removed sequence affected only the specific genome but not the overall analysis.

### 2.5 Genomic Context and Transposable Element Analysis

For every validated gene-genome pair, we extracted the downstream intergenic region. Analysis was restricted to genes mapping to a single chromosome and strand, with a gene body length under 5 Mb.

#### 2.5.1 Region Quality Control

We quantified the downstream region using four window sizes: Full Intergenic – up to the next Coding DNA Sequence (CDS); 10 kb; 50 kb; 100 kb. To avoid artifacts from poor quality of the assembly, we excluded regions with more than 10% gaps (N-content). Furthermore, we required the entire chromosome to have at least 20% MGE coverage by length (assessed by RepeatMasker v4.2.1 [15]) to ensure adequate identification of the insertions.

#### 2.5.2 MGE Quantification:

For each valid window, we calculated the total length occupied by sense and anti-sense MGEs. The “Promoter Hijacking” signal was quantified as the bias toward anti-sense insertions relative to the upstream protein coding gene.

### 2.6 Identification of Anchor Candidates

We employed Density-Based Spatial Clustering of Applications with Noise (DBSCAN) [16] to identify genes with a conserved architecture of the 3’ UTR. We utilized two distinct clustering modes depending on the genomic context.

#### 2.6.1 Expansion based model

For most genomic regions we used a model based on inflation of the intergenic space by strand-purified MGE insertions. We mapped genes into a 2D space as defined by: X is $L_{DS}/\text{Median}\left(L_{DS}\right)$ and $Y=2\times \left(L_{MGE}^{AS}-L_{MGE}\right)/L_{DS}$, where $L_{DS}$ is downstream length, $L_{MGE}$ length of MGE insertions in sense and $L_{MGE}^{AS}$ in anti-sense orientation. Clustering parameters were set to ε=0.2 and minimum points to 10. A cluster was flagged as an “Anchor” candidate if it met the following criteria: Mean Antisense Bias (Y) at least 0.75; Median Downstream Length at least 25 Kb; Representation from at least 5 distinct genomes; Median Antisense MGE content in the first 10 kb of at least 4 Kb.

#### 2.6.2 Adaptive model for gene deserts

For genes located in sparse regions (more than 50% of the analyzed genomes showing at least 50 Kb downstream space), the length metric loses the discriminative power. We adapted the algorithm as follows: X is a binary indicator ($L_{DS}$ less than 50 Kb; Y is the local strandedness bias within the first 50 Kb of the downstream intergenic space; ε=0.1 requiring tighter clustering.

#### 2.6.3 Phylogenetic consistency filter

Finally, we applied a phylogenetic consistency filter requiring at least one taxonomic family to show at least two times of increased odds of membership in the anchor cluster, ensuring that the cluster does not consist of unrelated genomes selected by random noise effects.

### 2.7 Phylogenetic trees construction

To construct the phylogenetic trees we used taxize [17] to arrange the taxa into the orders. To visualize average numerical values for such downstream genomic space length and strandedness across taxonomic ranks we developed a custom recursive algorithm going from the common root, e.g. Eutheria, to the tips of the tree and back for each rank averaging the values among the immediate children. This approach removes the bias caused by higher representation of well-studies animals with multiple subspecies. The result was repackaged into a temporary JSON file and forwarded to ggtree [18] for visualization.

### 2.8 Hypothesis validation

#### 2.8.1 sRNA seq data processing

We downloaded publicly available feline short RNA sequencing data [19] from SRA NCBI (https://www.ncbi.nlm.nih.gov/sra). Samples were merged together based on the sex and age into “Adult testis”, “Juvenile testis”, and “Prepubertal ovary” to increase the coverage depth. Adapters were trimmed using cutadapt [20] and the reads were mapped using STAR [21] on F.catus_Fca126_mat1.0 reference genome assembly [22] and the following parameters “--outFilterMismatchNmax 1 --alignEndsType EndToEnd --alignIntronMax 1 --alignIntronMin 1 --outFilterMultimapNmax 100 --alignSJDBoverhangMin 999”.

#### 2.8.2 mRNA seq data processing

We downloaded publicly available RNA seq data for *S. coeruleoalba* testis [23] and lung [24]. Both datasets were mapped to mSteCoe1.1 reference genome [24]. Using the following parameters “--outFilterMultimapNmax 1” for the testis, and “--outFilterMultimapNmax 1 --alignIntronMax 10000 --alignMatesGapMax 100000” for the lung. In both cases, only the reads corresponding to the plus strand transcription were loaded into the genome browser.

#### 2.8.3 Tissue expression bias analysis

We hypothesized that co-opted anchor genes exhibit testis-biased expression. We obtained Transcripts Per Million (TPM) data from the GTEx database (GTEx_Analysis_2025-08-22_v11_RNASeQCv2.4.3_gene_median_tpm.gct) covering 68 tissue types. Expression values were Z-score normalized per gene. We compared the testis Z-scores of flagged “anchor” candidates against the background set using a one-sided Welch’s t-test since our hypothesis involves higher expression in testis.

## 3 Results

### 3.1 Synteny-Aware Orthology Mapping Across 223 Mammalian Genomes

Our primary challenge was to identify true orthologs across a diverse set of Eutherian genomes, many of which are fragmented or annotated with varying quality. We based identification of protein coding genes on amino acid sequence similarities within a set of nine representative mammalian species: human (*H. sapiens*), mouse (*M. musculus*), dog (*C. lupus familiaris*), cat (*F. catus*), cow (*B. taurus*), camel (*C. dromedarius*), elephant (*L. Africana*), pig (*S. scrofa*), whale (*B. musculus*). First, we used ENSEMBL ortholog annotation to identify a set of 9847 genes which have exactly one ortholog between human and rest of the representative species. After the testing (see Methods), we reduced the library to 9754 HMM probes and used it to perform a global screen of 223 mammalian assemblies.

#### 3.1.1 Training of Synteny Rules:

We defined genomic “transaction” as the set of genes co-occurring within a 5 Mb window. Using high-quality annotations from nine representatives, we mined association rules using the Apriori algorithm [25]. Parameters were set to capture robust linkage: minimum support of 1.25·10^−5^, minimum confidence of $1-\frac{1}{N_{\text{species}}+1}$, maximum length of 3 items (the target with 2 neighbors). This generated an initial set of 47’175’853 rules.

To efficiently filter for evolutionary stability, we added a validation step by retaining only the rules that were satisfied (neighbor presence confirmed) in at least 8 of the 9 representative species. To facilitate the evaluation we developed a sparse matrix-based approach and converted the LHS and RHS matrices returned by the Apriori algorithm into binary form – the values stays 0 if the original value is 0, otherwise the value becomes 1. The result was summed as $R=LHS^{\text{binary}}+RHS^{\text{binary}}$, which is also binary. For each of the representative genome we constructed a binary transaction matrix $T^{\text{specie}}$ where the rows and the columns are genes with value 1 encoding presence of a transaction involving both genes. Multiplication of $R\times T^{\text{specie}}=S^{\text{specie}}$ leads to a matrix of (number of rules, number of genes) size. For each row of the $S^{\text{specie}}$ matrix, we took the maximum value corresponding to the number of genes that support the rule in a given representative genome. The rule is considered compliant if the number of genes is greater or equal to the number of genes mentioned in the rule. This reduced the dataset to 3’840’354 robust synteny rules, which were stored in a relational SQLite database for faster access.

The application of association rule mining—leveraging 3.8 million high-confidence synteny rules – rescued orthologs that would have been ambiguous or lost by sequence similarity alone. We successfully identified 9518 unique orthologous genes (97% of the probe set). Median number of valid hits recovered for a gene is 180 of the 223 analyzed genomes. The median number of genes recovered per genome was 9402, confirming the robustness of the pipeline even for non-model organisms.

### 3.2 Identification of 42 Conserved “Anchor” Candidates

To identify host genes that have been co-opted for piRNA cluster generation, we analyzed the downstream genomic landscape of every identified ortholog set. We searched for the specific “Promoter Hijacking” signature, which included a conserved proteincoding gene followed by a lineage-specific accumulation of antisense MGE insertions. Using a 2D clustering algorithm (DBSCAN) [16], we identified 42 genes that consistently displayed this architectural phenotype across multiple species. Genomic space expansion-based model flagged 31 genes depending upon the downstream region length and antisense MGE bias signatures. Adaptive model tracking local density of MGE insertions flagged 11 genes located in gene-sparse regions based on high local density of antisense MGEs within the first 50 kb downstream.

Notably, our unbiased screen independently recovered two known mammalian genes driving piRNA; these genes encode *NOPCHAP1* [10] (Fig S1) and *LONRF2* [26] (Fig S2). The rediscovery of these established genes validates the sensitivity of our model to detect genuine piRNA-generating transcripts.

### 3.3 Validation of Novel Anchors: *ORC6* in Felidae

**Fig. 1:**
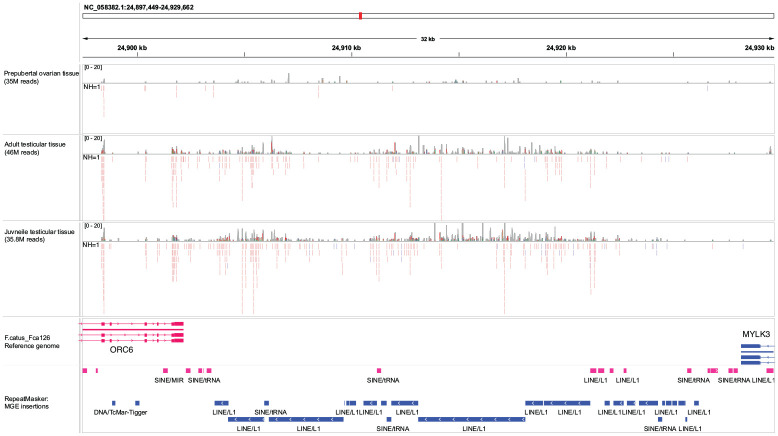
Genome browser view of feline *ORC6* downstream region. Coverage tracks include all alignments. Only unique mapping alignments are geometrically visualized

Among the top new candidates is *ORC6* (Origin Recognition Complex Subunit 6), a gene essential for DNA replication (Fig S3). In the *Felidae* lineage (cats), *ORC6* is immediately followed by a region of antisense MGE insertions. To confirm whether this architecture results in piRNA production, we analyzed small RNA-seq data from *Felis catus* testes (juvenile and adult) and ovaries as shown in Figure 1. Small RNA reads mapped to the *ORC6* coding sequence in all three libraries, consistent with normal mRNA turnover. The predicted region of “secondary function” region showed a male-specific expression pattern with dense small RNA production extending into the downstream intergenic space. The signal was most intense in juvenile males. This corresponds to the higher proportion of spermatogonia in prepubertal testes, suggesting that *ORC6* drives a pre-pachytene piRNA cluster essential for early stages of spermatogenesis.

### 3.4 Validation of elongated transcription: *SAYSD1* in Cetaceans

**Fig. 2:**
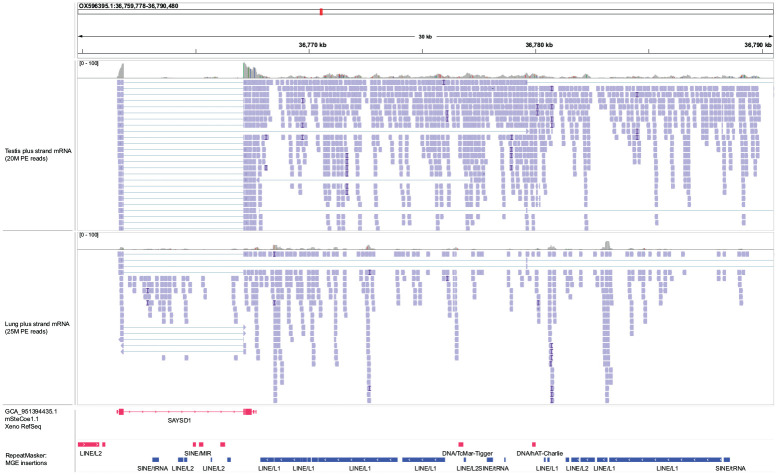
Genome browser view of the plus strand mRNA seq data from Stiped Dolphin testis (upper) and lung (lower) in the vicinity of the *SAYSD1* gene

In Cetaceans (whales and dolphins), the gene *SAYSD1* was flagged for a downstream expansion (Fig S4). To test its expression, we analyzed testicular mRNA-seq data from the striped dolphin (*Stenella coeruleoalba*). The alignment revealed a continuous, unannotated 23 kb 3’ UTR of *SAYSD1* (Figure 2), confirming the mechanistic basis of our model. Thus, the host gene promoter drives the transcription of a long, multikilobase precursor that traverses the downstream MGE-rich region and provides the necessary substrate for piRNA biogenesis.

### 3.5 Global Functional Analysis: Testis-Biased Expression

**Fig. 3:**
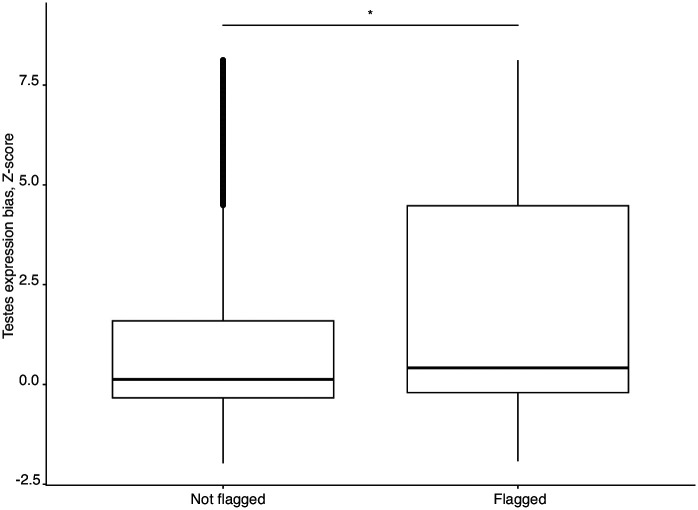
Z-scores of testis gene expression for not flagged (N=9476) and flagged (N=42) genes. The one-sided p-value is 0.02

If the “Promoter Hijacking” hypothesis is correct, the co-opted host genes must be highly expressed in the germline to support their secondary immune function. We tested this prediction using human tissue expression data from the GTEx project (68 tissues). We compared the tissue-specificity of our 42 flagged candidates against the background gene set. The anchor candidates showed a significant enrichment for testis-specific expression: the flagged genes have the mean Z-score = 2.06 (highly enriched in testis), and the background genes Z-score = 1.07 with p-value 0.02 (Figure 3). This result supports an evolutionary model where the piRNA machinery opportunistically targets host genes that are already transcriptionally active in the testis, “hijacking” their reliable promoters to drive the rapid evolution of genomic defense.

## 4 Discussion

This study addresses a fundamental paradox in genome evolution: how does the highly conserved piRNA immune system maintain a stable defense when its memory banks—piRNA clusters—are composed of rapidly mutating mobile genome elements? We propose and test a “Promoter Hijacking” model, where the genome co-opts stable, protein-coding host genes as regulatory anchors. By extending transcription beyond the canonical polyadenylation site and into downstream intergenic spaces, the host gene provides a reliable germline promoter to drive the expression of volatile, antisense MGE “payloads”. This mechanism allows for the rapid evolution of immune memory without requiring the *de novo* creation of new regulatory elements.

By analyzing these architectures across diverse mammalian lineages, we sought to identify genes that have transitioned from simple protein-coding units to dual-function loci supporting both normal cellular functions and genomic immunity. Our analysis reveals that piRNA clusters are not merely stochastic genomic accidents but are systematically tethered to essential host genes. The preference for genes with high testis-biased expression indicates that the evolution follows a path of least resistance, repurposing established germline transcriptional programs. This architectural coupling provides a robust solution to the arms race against MGEs: the “anchor” remains immutable under purifying selection, whereas the “payload” remains plastic, allowing the host to capture and silence new genomic threats while concurrently preserving the stability of the locus.

### 4.1 Limitations

Our study identifies high-confidence candidate genes, yet it is not intended to be a comprehensive list of all mammalian piRNA “anchoring” genes. For instance, *BAG2* – a gene coupled to a piRNA cluster in pigs [26] – was not captured due to the limited number of Suidae assemblies available to satisfy our taxonomic representation thresholds. Similarly, loci such as *Zim2* [9] were excluded because they are not protein-coding genes. The clustering algorithm itself is not precise and requires manual inspection of the flagged genes to remove false positive results.

Finally, the available biological evidence remains a limitation; the current “gold standard” for piRNA validation – immunoprecipitated PIWI proteins bound to small RNA – is unavailable for the vast majority of non-model species. The small RNA feline samples are the next best alternative, whereas the dolphin samples consist of mRNA which potentially can be processed into piRNA.

## Supplementary Material

**Supplementary information.** Supplementary figures S1-S4 contain signatures of piRNA clusters downstream of *NOPCHAP1*, *LONRF2*, *ORC6*, *SAYSD1* overlapped onto Eutherian phylogenetic trees.

Supplementary Files

This is a list of supplementary files associated with this preprint. Click to download.

- EvomanuscriptSUPPL.pdf

## Data Availability

This study did not generate new datasets. Custom code used for data analysis is available in GitHub (https://github.com/NEI-NNRL/piRNA_evolution/).
